# Overexpression of *Nrdp1* in the Heart Exacerbates Doxorubicin-Induced Cardiac Dysfunction in Mice

**DOI:** 10.1371/journal.pone.0021104

**Published:** 2011-06-27

**Authors:** Yuan Zhang, Yu-Ming Kang, Cui Tian, Yong Zeng, Li-Xin Jia, Xu Ma, Jie Du, Hui-Hua Li

**Affiliations:** 1 Department of Pathology, Institute of Basic Medical Sciences, Chinese Academy of Medical Sciences and Peking Union Medical College, Beijing, China; 2 Department of Physiology and Pathophysiology, Xi'an Jiaotong University School of Medicine, Xi'an, China; 3 The Key Laboratory of Remodeling-Related Cardiovascular Diseases, Department of Pathology, School of Basic Medical Sciences, Capital Medical University, Beijing, China; 4 Department of Cardiology, Peking Union Medical Hospital, Beijing, China; 5 The Key Laboratory of Remodeling-Related Cardiovascular Diseases, Laboratory of Vascular Biology, Beijing Institute of Heart Lung and Blood Vessel Diseases, Beijing Anzhen Hospital Affiliated to Capital Medical University, Beijing, China; 6 Department of Genetics, National Research Institute for Family Planning, Beijing, China; Cleveland Clinic, United States of America

## Abstract

**Background:**

Cardiac cell death and generation of oxidative stress contribute to doxorubicin (DOX)-induced cardiac dysfunction. E3 ligase *Nrdp1* plays a critical role in the regulation of cell apoptosis, inflammation and production of reactive oxygen species (ROS), which may contribute to heart failure. However, the role of *Nrdp1* in DOX-induced cardiac injury remains to be determined.

**Methods and Results:**

We examined the effect of *Nrdp1* overexpression with DOX treatment in rat neonatal cardiomyocytes and mouse heart tissue. Cardiomyocytes were infected with adenovirus containing GFP (Ad-GFP), *Nrdp1* wild-type (Ad-*Nrdp1*) or the dominant-negative form of *Nrdp1* (Ad-Dn-*Nrdp1*), then treated with DOX for 24 hr. DOX treatment increased cell death and apoptosis, with Ad-*Nrdp1* infection enhancing these actions but Ad-Dn-*Nrdp1* infection attenuating these effects. Furthermore, 5 days after a single injection of DOX (20 mg/kg, intraperitoneally), *Nrdp1* transgenic mice (TG) showed decreased cardiac function and increased apoptosis, autophagy and oxidative stress as compared with wild-type (WT) mice (*P*<0.01). Survival rate was significantly lower in *Nrdp1* TG mice than in WT mice 10 days after DOX injection (*P*<0.01).

**Conclusions/Significance:**

These results were associated with decreased activation of Akt, extracellular signal-regulated kinase 1/2 (ERK1/2) and signal transducer and activator of transcription 3 (STAT3) signaling pathways. *Nrdp1* may be a key mediator in the development of cardiac dysfunction after DOX treatment and associated with inhibition of Akt, ERK1/2 and STAT3. *Nrdp1* may be a new therapeutic target in protecting against the cardiotoxic effects of DOX.

## Introduction

Doxorubicin (DOX), an anthracycline antibiotic, is one of the most potent anti-neoplastic agents used to treat various solid and hematogenic tumors [1]. Cardiotoxicity leading to congestive heart failure is the major factor limiting the clinical use of DOX [1], [2]. Despite intensive investigations of DOX-induced cardiotoxicity, the molecular mechanisms underlying DOX-induced cardiac injury and dysfunction have not been completely elucidated. Emerging evidence from animal and human studies indicates that DOX-induced cardiomyopathy is mainly caused by increased production of reactive oxygen species (ROS), inflammation, apoptotic cell death and vacuolization of myocardial cells, which are the typical changes in DOX-induced heart failure [3], [4].

*Nrdp1* (also known as FLRF or RBCC) is a newly defined RING finger E3 ubiquitin ligase that has important roles in regulating cell growth, apoptosis, and oxidative stress in various cell types [5], [6], [7], [8], [9], [10]. Accumulating evidence indicates that *Nrdp1* promotes ubiquitination and degradation of the epidermal growth-factor receptor family member ErbB3, the gigantic (530 kDa) inhibitor-of-apoptosis protein BRUCE, and Parkin, which have been implicated in the pathology of numerous disorders, including heart valve formation, cancer, Parkinson's disease, and endotoxin shock [5], [6], [7], [8], [9], [10], [11]. Recently, we demonstrated that cardiac-specific overexpression of *Nrdp1* can promote cardiac myocyte apoptosis induced by ischemia-reperfusion (I/R) injury in transgenic mice, and inhibition of endogenous *Nrdp1* in cardiomyocytes protects against I/R-triggered apoptosis *in vitro* [12]. However, the functions of *Nrdp1* in DOX-induced cardiomyopathy and its underlying mechanism(s) have not been investigated.

On the basis of our prior findings, we postulated that increased *Nrdp1* levels would exacerbate DOX-triggered cardiotoxicity. We investigated the role of *Nrdp1* in DOX-induced cardiomyocyte death and production of oxidative stress, as well as mouse survival and left ventricle (LV) function. We studied the activation of Akt, extracellular signal-regulated kinase 1/2 (ERK1/2), and signal transducer and activator of transcription 3 (STAT3) pathways in the process.

## Results

### Effect of DOX on the expression of *Nrdp1* in rat neonatal cardiomyocytes and mouse heart

**Figure 1:**
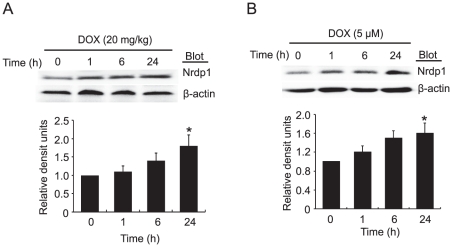
DOX stimulates *Nrdp1* protein expression. Time-course of the protein expression of *Nrdp1* in mouse hearts (n = 3) after intraperitoneal (ip) injection of 20 mg/kg DOX (A) or in neonatal rat cardiomyocytes after 0.5 µM DOX treatment (B). β-actin was used as a loading control. Quantitative analysis is in bottom panels (n = 3 experiments for each time points). *P<0.05 vs 0 hour.

To investigate the role of *Nrdp1* in cardiac injury in response to DOX treatment, we first examined the expression of endogenous *Nrdp1* in mouse heart tissues at different times; *Nrdp1* protein level was time-dependently upregulated with intraperitoneal (ip) injection of 20 mg/kg DOX (Figure 1A). This result was further confirmed in rat neonatal cardiomyocytes after 0.5 µM DOX treatment (Figure 1B). DOX treatment simulating *Nrdp1* expression in cardiomyocytes suggested that *Nrdp1* might be involved in DOX-induced cardiotoxicity.

### DOX-induced cardiomyocyte death is suppressed by dominant-negative *Nrdp1*

**Figure 2:**
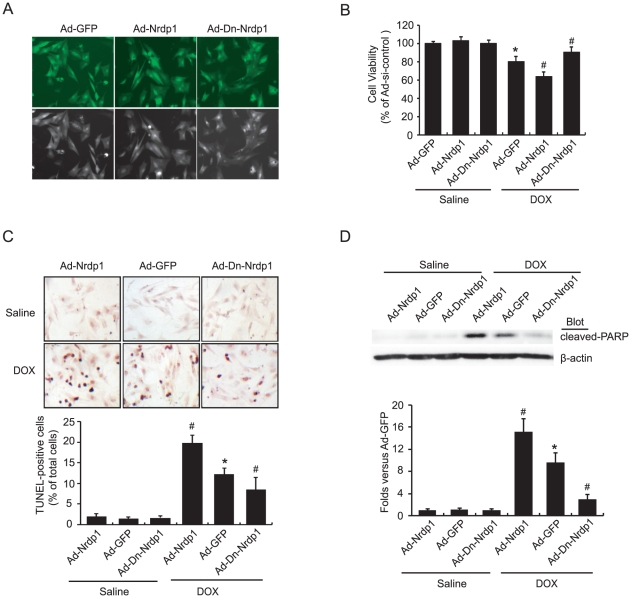
Effects of *Nrdp1* on DOX-induced cell death in rat neonatal cardiomyocytes. A. The infection efficiency of cardiomyocytes with Ad-GFP, Ad-*Nrdp1* and Ad-Dn-*Nrdp1* was visualized by green fluorescent protein (GFP) 24 hours later on fluorescence microscopy. (magnification, ×400). B. Cardiomyocytes were infected by with Ad-GFP, Ad-*Nrdp1* or Ad-Dn-*Nrdp1* and then treated with 5 µM DOX for 24 hr. Cell viability was measured by trypan blue exclusion assay. C. Apoptosis was detected by TUNEL assay. A representative field is shown for each condition (top panels). Quantitative analysis of TUNEL-positive cells from 3 independent experiments (bottom panels, magnification, ×200). D. Cardiomyocytes were infected and treated with DOX as in A. Western blot analysis of expression of cleaved poly ADP-ribose polymerase (PARP) protein (top panel). Quantitative analysis is in the bottom panel. *P<0.01 vs. Ad-GFP. ^#^ *P*<0.05 vs. Ad-GFP+DOX. Results are expressed as means ± SEM (n = 3).

To determine whether *Nrdp1* could affect DOX-induced cardiomyocyte death, rat neonatal cardiomyocytes were infected with Ad-GFP, Ad-*Nrdp1* or Ad-Dn-*Nrdp1* (lacking E3 ubiquitin ligase activity). The infection efficiency was >95% after 24 hr (Figure 2A). Infected cells were subsequently subjected to DOX (0.5 µM) or saline treatment. Cell viability, TUNEL-positive cells and level of cleaved poly ADP-ribose polymerase (PARP), an indicator of programmed cell death were similar among the groups after saline treatment (Figure 2B–D). At 24 hr after DOX treatment, cell viability was markedly decreased, and TUNEL-positive cells and cleaved PARP level were significantly increased in Ad-GFP-infected groups as compared with saline-treated groups. These effects were further enhanced with Ad-*Nrdp1* infection. In contrast, these alterations were markedly diminished with Ad-Dn-*Nrdp1* infection, which suggests that *Nrdp1* can promote DOX-induced cell death and apoptosis.

### *Nrdp1* overexpression enhances DOX-induced cardiac injury and apoptosis

**Figure 3:**
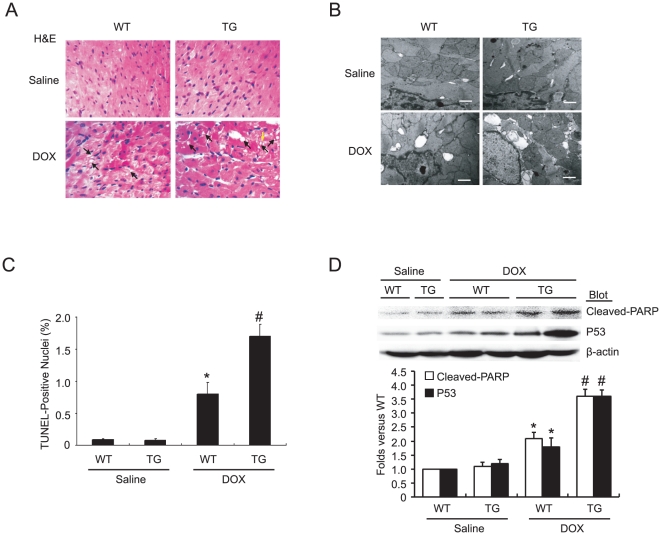
Cardiac-specific overexpression of *Nrdp1* aggravates DOX-induced cardiac injury and cardiomyocyte apoptosis. A. H&E staining of heart sections on day 5 after saline or DOX treatment (n = 4 per group). Arrows indicate representative vacuolization. Magnification ×200. B. Representative electron microphotographs of cardiac sections from wild type (WT) (left panel) and *Nrdp1* transgenic (TG) mice (right panel) treated with saline or 20 mg/kg DOX for 4 days (n = 3 per group). Arrows indicate cytoplasmic vacuolization. Scale bars = 1 µm. C. Quantitative results of cardiomyocyte apoptosis detected by TUNEL assay (n = 4 per group). D. Western blot analysis of protein level of cleaved PARP and p53 in WT and *Nrdp1* TG mice treated with saline or DOX (n = 5 per group). Quantitative analysis is in the bottom panels. *P<0.01 vs WT+saline mice; ^#^ *P*<0.01 vs WT+DOX mice.

To confirm whether *Nrdp1* overexpression affects DOX-induced cardiac injury and apoptosis *in vivo*, we created transgenic mice (TG) overexpressing *Nrdp1* under the control of the cardiac-specific α-myosin heavy chain (α-MHC) promoter. *Nrdp1* TG hearts showed a 2.8-fold increase in total (transgenic and endogenous) *Nrdp1* content as compared with WT hearts [12]. At 5 days after DOX injection, WT hearts showed focal cytoplasmic vacuolization, a hallmark of cell injury, which is consistent with previous reports [13], [14], and this alteration was greater in TG than WT hearts (Figure 3A). Electron microscopy further demonstrated severe vacuolar degeneration in DOX-treated *Nrdp1* TG hearts as compared with WT hearts (Figure 3B). Furthermore, the proportion of TUNEL-positive nuclei was 2.1-fold higher in DOX-treated TG than WT hearts (Figure 3C). Accordingly, the protein levels of cleaved PARP and p53 were markedly higher in DOX-treated WT than saline-treated WT mice, and the increased expression of these proteins was induced by 45% in DOX-treated TG hearts as compared with controls (Figure 3D). Thus, overexpression of *Nrdp1 in vivo* significantly increased DOX-induced cardiac injury and apoptosis.

### Effect of *Nrdp1* on DOX-induced autophagy

**Figure 4:**
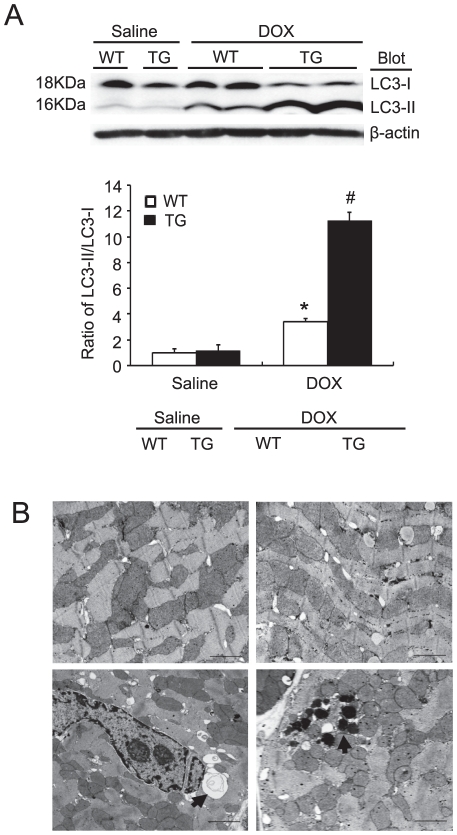
*Nrdp1* TG mouse hearts are more susceptible to DOX-induced autophagy. A. Western blot analysis of ratio of LC3-II to -I protein level from WT and *Nrdp1* TG heart tissue after saline or DOX treatment. A representative blot is shown for each condition (top panels). β-actin was used as a loading control. Histograms show relative intensity of the ratio of LC3-II to -I (n = 4 per group) (bottom panel). *P<0.05 vs WT+saline mice; ^#^ *P*<0.05 vs WT+DOX mice. B. Representative electron microphotographs of cardiac sections from WT (left panel) and *Nrdp1* TG mice (right panel) treated with 20 mg/kg of DOX for 4 days (n = 3 per group). Arrows indicate autophagic vacuoles and electron-dense lysosomes. Scale bars = 1 µm.

A recent study demonstrated that DOX induces autophagy in cardiomyocytes, and increased autophagy promotes DOX-induced cardiomyocyte death [15]. We thus examined whether overexpression of *Nrdp1* could affect DOX-induced autophagy in the mouse heart. Microtubule-associated protein light 1 chain 3 (LC3), a mammalian homologue of yeast Atg8, is processed from LC3-I (18 kDa) to -II (16 kDa) and incorporated into autophagic vacuoles, and the quantification of the ratio of LC3-II to -I is a good marker of autophagy induction [16]. Western blot analysis showed that the baseline ratio of LC3-II to -I was similar in WT and *Nrdp1* TG hearts. After 24 hr of DOX injection, the ratio was significantly higher in DOX-treated than saline-treated WT hearts and was greater after DOX treatment in TG than WT hearts (Figure 4A). Consistent with the immunoblotting results of LC3 expression, electron microscopy after DOX treatment revealed a marked accumulation of vacuoles in WT cardiomyocytes, which was exaggerated in TG cardiomyocytes, most of which were electron-dense lysosomes (Figure 4B). The ultrastructure of cardiomyocytes was not affected by saline treatment (Figure 4B). Thus, overexpression of *Nrdp1 in vivo* could increase DOX-induced autophagy in the mouse heart.

### Effect of *Nrdp1* on DOX-induced oxidative stress

**Figure 5:**
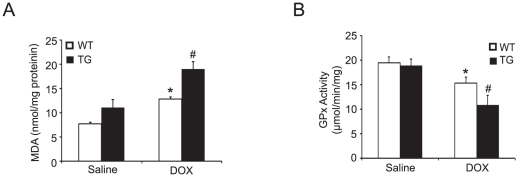
*Nrdp1* overexpression increases DOX-induced oxidative stress. Malondialdehyde (MDA) level (A) and glutathione peroxidase (GPX) activity (B) were measured to estimate extent of lipid peroxidation and antioxidation in heart homogenates from WT and *Nrdp1* TG mice (n = 5 per group) treated with saline or DOX. *P<0.05 vs WT+saline mice; ^#^ *P*<0.05 vs WT+DOX mice.

Previous study showed that *Nrdp1* plays an important role in ROS generation in SH-SY5Y cells [7]. We thus compared the levels of 2 oxidative stress markers, malondialdehyde (MDA) and glutathione peroxidase (GPX) in heart tissues from WT and *Nrdp1* TG mice. MDA level and GPX activity did not differ between WT and TG hearts after saline injection (Figure 5A and B). After 4 days of DOX administration, MDA level was increased and GPX activity decreased in WT and TG hearts as compared with saline-treated hearts (*P*<0.05). Moreover, these changes in MDA level and GPX activity were greater in TG than WT hearts (*P*<0.05).

### Cardiac-specific expression of *Nrdp1* promotes DOX-induced cardiac dysfunction

**Figure 6:**
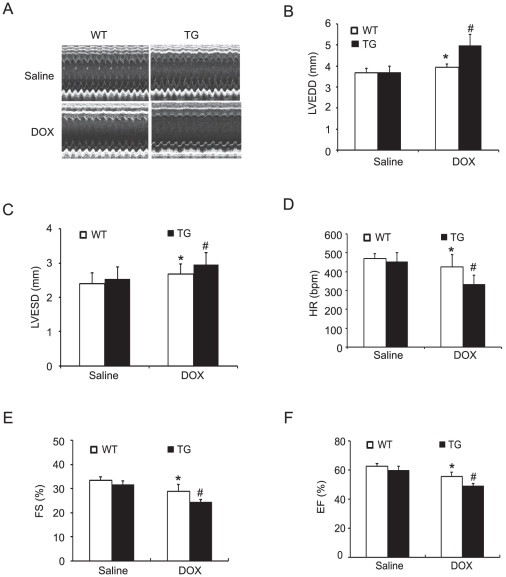
Overexpression of *Nrdp1* reduces cardiac function after administration of DOX. (A) Representative M-mode echocardiograms of WT and *Nrdp1* TG mice treated with saline or DOX. Quantitative group data for echocardiographic measurements: (B) HR; (C) LVEDD; (D) LVESD; (E) FS; and (F) EF. Data were from 10 mice in each group. *P<0.05 vs WT+saline group, ^#^ *P*<0.05 vs WT+DOX group. Abbreviations are defined in the text.

Given the marked disparity of cardiomyocyte injury and cell death between WT and *Nrdp1* TG mice after DOX administration, we assessed cardiac contractile function by *in vivo* echocardiography. Figure 6A shows representative echocardiograms after saline or DOX administration in WT and TG mice. Systolic, diastolic, and cardiac function did not differ between WT and TG mice after saline injection. However, at 4 days after DOX injection (20 mg/kg), LVEDD and LVESD were greater in WT alone than saline-treated WT hearts, and the increased LVEDD and LVESD was significantly higher in TG than WT hearts, so DOX-induced LV dilatation was increased in *Nrdp1* TG mice (Figure 6B, C). Importantly, DOX markedly reduced HR, FS and LVEF in both WT and TG mice (P<0.01), with a greater decrease in TG than WT mice (P<0.01) (Figure 6D,E,F). Thus, overexpression of *Nrdp1* in the mouse heart exacerbates the DOX-induced cardiac dysfunction.

### Survival analysis

**Figure 7:**
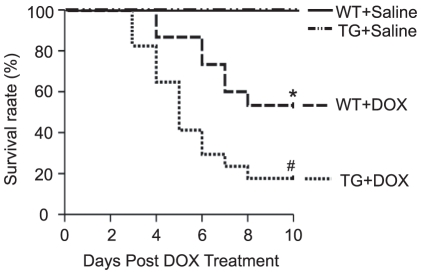
Survival rate. Ten-day survival was analyzed in WT and *Nrdp1* TG mice treated with DOX (20 mg/kg). Survival was analyzed by the Kaplan-Meier approach and the log-rank test (*n* = 15 per group). *P<0.05 vs WT+saline mice; ^#^ *P*<0.05 vs WT+DOX mice.

Ten days after saline treatment, WT and *Nrdp1* TG mice did not differ in survival. Ten days after DOX treatment, the survival was significantly lower in TG than WT mice (17% vs. 53%, P<0.01). The survival was markedly lower in DOX-treated than untreated WT controls (53% vs 100%, P<0.01) (Figure 7). Therefore, overexpression of *Nrdp1* in the heart reduced prolonged survival.

### Effect of *Nrdp1* on activation of Akt, ERK1/2 and STAT3 signaling pathways after DOX treatment in rat neonatal cardiomyocytes

**Figure 8:**
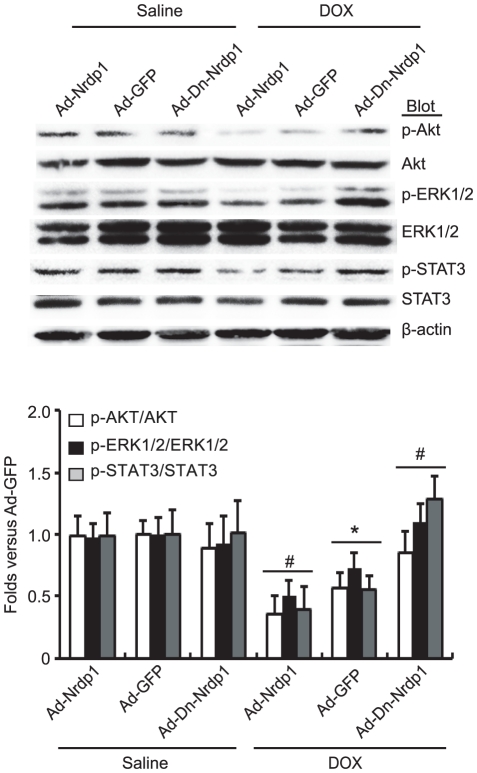
Effect of *Nrdp1* on the phosphorylation of Akt, ERK1/2 and STAT3 in rat neonatal cardiomyocytes. Rat neonatal cardiomyocytes were infected by with Ad-GFP, Ad-*Nrdp1* or Ad-Dn-*Nrdp1* and then treated with 0.5 µM DOX for 24 hr. Western blot analysis of protein levels of phosphorylated and total Akt, ERK1/2 and STAT3 (n = 4 per group, top panels). β-actin was used as a loading control. A representative blot is shown for each condition. Quantitative analysis is in the bottom panel. *P<0.05 vs. Ad-GFP+saline; ^#^ *P*<0.05 vs. Ad-GFP+DOX.

Three classes of signaling pathways, Akt, ERK1/2 and STAT3, are involved in DOX-induced cardiotoxicity [13], [17], [18], [19], [20], so we detected the activation of Akt, ERK/12 and STAT3 in neonatal rat cardiomyocytes by western blot analysis. The levels of phosphorylated Akt, ERK1/2 and STAT3 were similar among groups with saline treatment (Figure 8). At 24 hr after DOX treatment, the levels of phosphorylated Akt, ERK1/2 and STAT3 were decreased in Ad-GFP-infected cardiomyocytes, with the levels further lowered in *Nrdp1*-infected cells than in the GFP control. However, the levels were markedly alleviated in Ad-Dn-*Nrdp1*-infected cells as compared with Ad-*Nrdp1*-infected cells (Figure 8). Levels of total Akt, ERK1/2 and STAT3 did not differ among groups (Figure 8). Thus, *Nrdp1* may promote DOX-induced cardiomyocyte death via the Akt, ERK1/2, and STAT3-dependent signaling pathways.

## Discussion

*Nrdp1*, as an E3 liagse, has been extensively investigated for apoptosis and inflammation activity in cancer cells and other cell types [5], [6], [7], [8], [9], [10], [11]; however, the effect of *Nrdp1* on DOX-induced cardiotoxicity and underlying mechanisms has been less well studied. We revealed a critical role for *Nrdp1* in the development of cardiac injury after DOX treatment. Overexpression of *Nrdp1 in vivo* in mouse hearts and *in vitro* in cardiomyocytes enhanced DOX-induced cardiomyocyte apoptosis, autophagy and oxidative stress, thus resulting in more severe LV contractile dysfunction and mouse mortality after DOX injury. These changes were associated with inactivation of Akt, ERK1/2, and STAT3 signaling pathways. *Nrdp1* may be an important contributor in DOX-induced cardiac dysfunction.

Emerging evidence indicates that DOX induces cardiac injury via several mechanisms, including free radical generation, apoptosis and autophagy [15], [17], [21]. Cardiomyocyte apoptosis is one of the critical events in DOX-induced heart failure [17], [21]. *Nrdp1* promotes degradation of BRUCE/apollon, a 530-kDa membrane-associated inhibitor of apoptosis protein, thereby promoting apoptosis [9]. A recent study indicated that *Nrdp1* functions as a critical regulator of Toll-like receptor responses [10]. However, the role of *Nrdp1* in DOX-induced cardiac injury remains unclear. *In vitro* studies in neonatal cardiomyocytes showed increased expression of *Nrdp1* markedly enhancing DOX-induced cardiomyocyte apoptosis and the levels of cleaved PARP, an indicator of programmed cell death; infection with Dn-*Nrdp1* markedly attenuated this effect (Figure 2). Importantly, *Nrdp1* TG mice after DOX injection exhibited severe myocardial injury and a significant increase in TUNEL-positive cardiomyocytes and levels of cleaved PARP and p53 expression (Figures 2 and 3), thus leading to severe cardiac dysfunction and increased mortality in *Nrdp1* TG mice (Figures 6 and 7). These results suggest that extensive apoptosis might be the major contributor to the decreased survival and cardiac dysfunction observed in *Nrdp1* TG mice after DOX injury.

Autophagy, an intracellular bulk degradation process, is sensitive to physiological regulation, such as the supply and deprivation of nutrients. Sixteen proteins participate in the autophagy pathway in humans [16]. Besides increasing activities of cellular degradation pathways such as calpain and the ubiquitin proteasome system [22], [23]. DOX induces autophagy in cardiomyocytes [15]. Importantly, 3-methyadenine, an autophagy inhibitor, can reduce DOX-induced cardiomyocyte death, which suggests that autophagy activation may contribute to DOX-induced cardiotoxicity. Autophagy is often associated with apoptosis, which can act in partnership to coordinately induce cell death [16]. In the present study, we showed that DOX greatly increased the ratio of LC3-II to LC3-I, a marker of autophagy activation, and accumulation of vacuoles in cardiomyocytes. Overexpression of *Nrdp1* further enhanced these effects (Figure 4), which indicates that *Nrdp1* may mediate cardiac injury after DOX by inducing autophagy. Further studies are needed to address the exact molecular functions of *Nrdp1* in the activation of autophagy in the heart.

Oxidative stress can directly induce cell damage, cell apoptosis and autophagy [15], [21]. Several studies have demonstrated that agents such as statins that scavenge ROS protect against DOX-induced cardiac apoptosis [3], [24]. Furthermore, cardiac-specific overexpression of antioxidant genes, including manganese superoxide dismutase and catalase, protect mice against DOX-induced cardiac dysfunction [25], [26], [27]. More recently, increased expression of *Nrdp1* enhanced the production of ROS, whereas this effect was attenuated by knockdown of *Nrdp1* by siRNA in SH-SY5Y cells [7]. We found that DOX stimulated *Nrdp1* expression, so *Nrdp1* can be activated by oxidative stress such as DOX (Figure 1). Furthermore, after DOX injection, as compared with WT mice, *Nrdp1* TG mice showed a marked increase of MDA levels and decrease of GPX activity as indexes of oxidative stress (Figure 5). Thus, *Nrdp1* also plays a critical role in the generation of oxidative stress in the heart. However, the precise mechanisms involved in the interaction between *Nrdp1* and oxidative stress still need further investigation.

Several major signaling pathways, including Akt, ERK1/2, and STAT3 have been implicated in mediating DOX cardiotoxicity [13], [17], [18], [19], [20], [28]. Previous studies demonstrated that DOX treatment caused myocardial damage and reduced phosphorylation of Akt and ERK1/2 [28], whereas activation of Ras/MEK/ERK and Akt signaling can inhibit DOX-induced apoptosis and ameliorate DOX-induced congestive heart failure [13], [18], [20], [28]. Furthermore, mice with cardiac-specific deletion of STAT3 showed greater susceptibility to cardiac injury after DOX treatment [19]. For further insight into the mechanisms of *Nrdp1* in DOX-induced cardiotoxicity, we examined activation of these signaling pathways. Overexpression of *Nrdp1* decreased the phosphorylation of Akt, ERK/12, and STAT3, and the decreased kinase activity was reversed with Dn-*Nrdp1* infection in cardiomyocytes (Figure 8). Thus, *Nrdp1* has an important role in regulating the activation Akt, ERK1/2, and STAT3 signaling pathways in the heart during DOX injury.

### Conclusions

In the present study, we demonstrate that in response to DOX treatment, *Nrdp1* protein is upregulated in mouse hearts and cardiomyocytes, which results in increased cardiac apoptosis, autophagy, and generation of oxidative stress and thus cardiac injury and dysfunction. These effects are associated with inactivation of Akt, ERK1/2 and STAT3 signaling pathways. Therapeutic strategies such as pharmacological inhibition to block *Nrdp1* activation may help prevent cardiac apoptosis, autophagy and oxidative stress and thereby attenuate DOX cardiotoxicity.

## Materials and Methods

### Antibodies and reagents

The plasmids of mouse wild-type *Nrdp1* and dominant-negative form of *Nrdp1* (C34S/H36Q) were a kind gift from Dr. Xiaobo Qiu (College of Life Sciences, Beijing Normal University, China). Anti-*Nrdp1* (BETHYL Laboratories, Inc), anti-cleaved PARP, anti-total- and phospho-AKT (Ser473), -ERK1/2 (Thr202/Tyr204), -STAT3, Tyr705) and horseradish peroxidase-conjugated goat anti-mouse or anti-rabbit IgG secondary antibody were from Cell Signaling Technology. Anti-light 1 chain 3 (anti-LC3) was from MBL International Operation; anti-p53 and anti-β-actin were from Santa Cruz Biotechnology. Doxorubicin (DOX) and other reagents were from Sigma-Aldrich.

### Cell culture and adenoviral constructs

Neonatal rat cardiomyocytes were isolated from 1-day-old Sprague-Dawley rats by enzymatic disassociation [29]. Recombinant adenoviruses expressing green fluorescent protein (GFP) alone (Ad-GFP), *Nrdp1* (Ad-*Nrdp1*) and the dominant-negative form of *Nrdp1* (C34S/H36Q) (Ad-Dn-*Nrdp1*) driven by the cytomegalovirus promoter were generated by use of AdEasy (MP Biomedicals Inc.) [12], [29]. Twenty-four hours after plating, cells were infected with Ad-GFP, Ad-*Nrdp1*, or Ad-Dn-*Nrdp1* for 24 hr and then treated with 0.5 µM DOX for the indicated times. Cell viability was determined by Trypan blue exclusion assay [30].

### Animals and treatments

*Nrdp1* transgenic mice (TG) were generated and characterized as described [12], [29]. Male wild-type (WT) and *Nrdp1* transgenic (TG) mice in the same C57BL/6 background were 8 to 10 weeks old. WT (n = 106) and *Nrdp1* TG (n = 94) mice were randomly assigned to the control group or DOX-treated group. DOX (20 mg/kg) was administered by intraperitoneal (ip) injection as described [13]. Control mice received injections of saline at a comparable volume. All procedures were approved by and performed in accordance with the Animal Care and Use Committee of Capital Medical University (20090916). The investigation conformed to the Guide for the Care and Use of Laboratory Animals published by the US National Institutes of Health (NIH, 1996).

### Echocardiography

Male WT and *Nrdp1* TG mice (n = 10 per group) were lightly anesthetized with tribromoethanol (0.25 mg/g ip). Four days after DOX or vehicle (saline) injection, mice underwent high-resolution micro-ultrasonography (Vevo 770, VisualSonics Inc., Toronto, Canada) [29]. The left ventricular end diastolic diameter (LVEDD),left ventricular end systolic diameter (LVESD), LV fractional shortening (LVFS), LV ejection fraction (LVEF) and heart rate (HR) were measured. All variables were measured in the parasternal long-axis view over at least 5 consecutive cardiac cycles and averaged from at least 2 measurements. LVFS was calculated as [(LVEDD−LVESD)/LVEDD]×100.

### Morphological examination and TUNEL assay

Hearts were fixed with 10% formalin and embedded in paraffin. Heart sections (thickness, 5 µm) were stained with hematoxylin and eosin (H&E). Sections were also examined for apoptotic cardiomyocytes by TdT-mediated dUTP Nick-End Labeling (TUNEL) assay (TUNEL fluorescence FITC kit, Roche, USA) [31]. DAPI reagent was used to counterstain nuclei. The percentage of TUNEL-positive myocytes was determined by counting 10 random fields per section under a microscope (Leica, Germany).

### Electron microscopy

Ultrastructural injury in cardiac tissues of mice (n = 3 per group) treated with vehicle (saline) or DOX for 5 days was evaluated by electron microscopy. Heart tissue was cut into 1 mm^3^ pieces and immersion-fixed overnight in phosphate buffered 2.5% glutaraldehyde (pH 7.4), postfixed for 1 hr with 1% osmium tetroxide, dehydrated through a graded ethanol series, and embedded in Epon medium. Ultrathin sections (60–70 nm) were stained with uranyl acetate and lead citrate and observed under a JEOL 100-CX transmission electron microscope.

### Measurement of lipid peroxidation and antioxidant enzymes in the left ventricle

LV malondialdehyde (MDA) level as an indicator of lipid peroxidation was measured by use of the commercially available colorimetric assay kit (Nanjing Jiancheng Bioengineering Inst., China). Antioxidant enzyme was measured by assay of glutathione peroxidase (GPX) (Calbiochem).

### Western blot analysis

Western blot analysis was as described [29]. Protein samples from cardiomyocytes or heart tissues were separated by SDS-PAGE, transferred onto immobilon-P membrane (Millipore), then incubated with primary and secondary antibodies. Relative protein levels were normalized to that of β-actin.

### Statistical analysis

Data are presented as mean ± SEM. Comparison between groups involved Student's *t* test or one-way ANOVA. Survival after DOX injection in mice was analyzed by the Kaplan-Meier method and compared by a log-lank test. A *P*<0.05 was considered statistically significant.
