# Correction: Overexpression of *Nrdp1* in the Heart Exacerbates Doxorubicin-Induced Cardiac Dysfunction in Mice

**DOI:** 10.1371/journal.pone.0267515

**Published:** 2022-04-19

**Authors:** Yuan Zhang, Yu-Ming Kang, Cui Tian, Yong Zeng, Li-Xin Jia, Xu Ma, Jie Du, Hui-Hua Li

There is an error in the β-actin western blot panel in Fig 4A in this article [1]. Specifically, the panel for β-actin is incorrect and has been duplicated from the β-actin western blot panel in Fig 2D.

**Fig 4:**
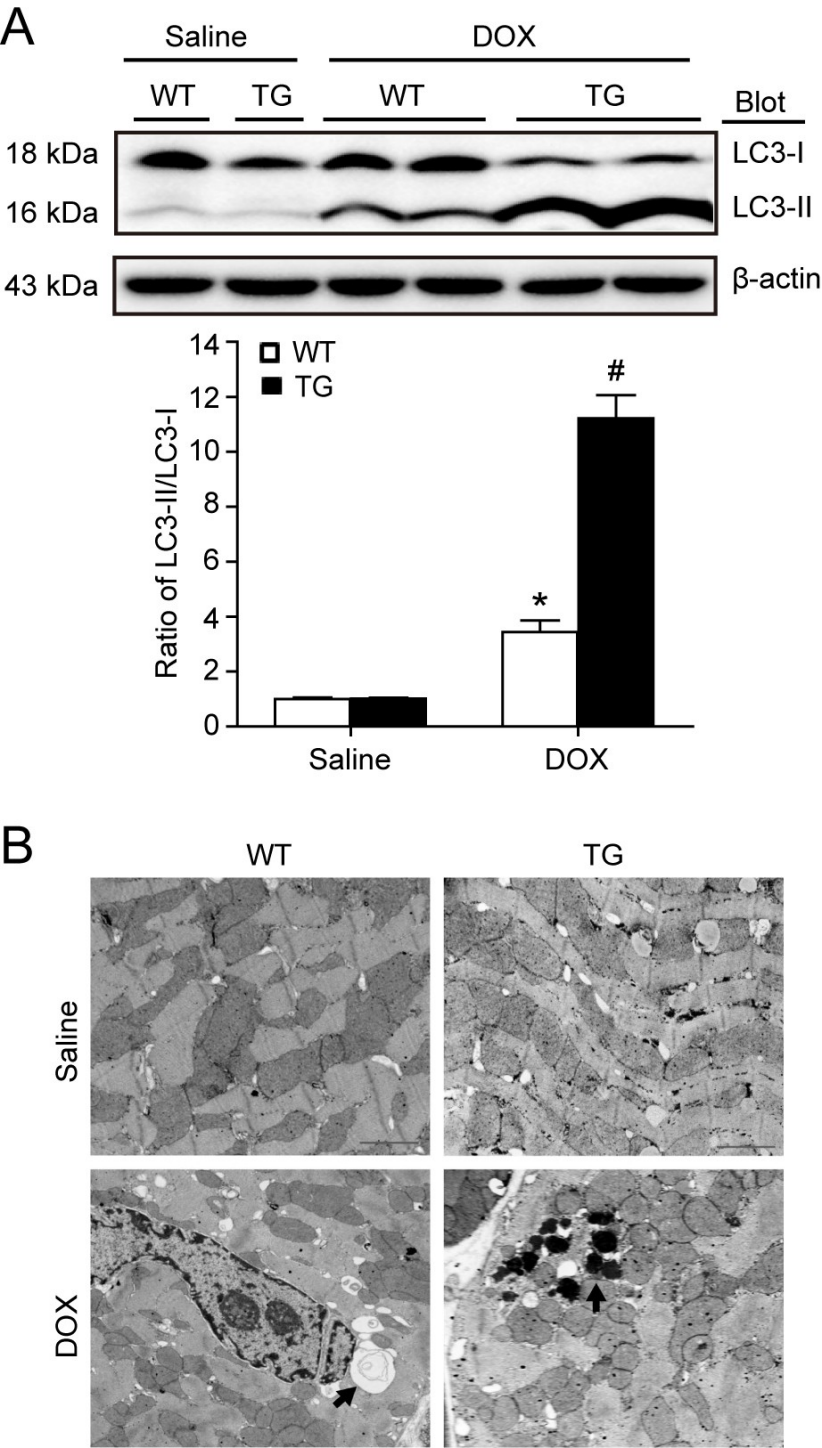
*Nrdp1* TG mouse hearts are more susceptible to DOX-induced autophagy. A. Western blot analysis of ratio of LC3-II to -I protein level from WT and *Nrdp1* TG heart tissue after saline or DOX treatment. A representative blot is shown for each condition (top panels). β-actin was used as a loading control. Histograms show relative intensity of the ratio of LC3-II to -I (n = 4 per group) (bottom panel). *P<0.05 vs WT+saline mice; ^#^ *P*<0.05 vs WT+DOX mice. B. Representative electron microphotographs of cardiac sections from WT (left panel) and *Nrdp1* TG mice (right panel) treated with 20 mg/kg of DOX for 4 days (n = 3 per group). Arrows indicate autophagic vacuoles and electron-dense lysosomes. Scale bars = 1 μm.

Here the authors provide a revised version of Fig 4 in which the β-actin western blot panel has been replaced with the image from the original experiment. The chart in Fig 4A has also been replaced, and labels have been added to the electron micrographs in Fig 4B. Underlying data, including the original raw data and replication data from the original experiments, supporting the revised Fig 4A are in S1 and S2 Files. The authors measured the band intensities for the revised β-actin panel and revised the loading control values used in the quantitative analysis normalisation. The LC3-II and LC3-I band intensities were recalculated and normalised to the revised β-actin values, and are presented in the revised Fig 4.

The authors have clarified that they used the same β-actin control in Figs 2D and 4A. The actin blots and blots for the proteins of interest were obtained from different gels in Figs. 1, 3D and 4A and from the same gel in Fig 2D. The authors have also clarified that the gels in Figs 2D and 3D were cut during processing.

The Results statements describing Fig 2D say, “cleaved PARP level were significantly increased in Ad-GFP-infected groups as compared with saline-treated groups. These effects were further enhanced with Ad-*Nrdp1* infection. In contrast, these alterations were markedly diminished with Ad-Dn-*Nrdp1* infection.” These statements are hereby revised to: “Image data from triplicate experiments support the findings that there are higher levels of Cleaved PARP for all groups (Ad-*Nrdp1*, Ad-GFP, Ad-Dn-*Nrdp1*) in the presence of DOX as compared to saline treated controls, and there may be a modest reduction of Cleaved PARP in DOX-treated Ad-Dn-*Nrdp1* cells as compared to DOX-treated Ad-*Nrdp1* cells.”

The low resolution of the data file provided for Fig 2 precluded a detailed assessment as to the integrity of the images.

The authors apologize for the errors in the published article and state that the corrections to Fig 4 do not affect the validity of the data or the overall conclusions.

Underlying data supporting Fig 2D are in S3 and S4 Files and for other results in the published article are available from the corresponding author.

## Supporting information

S1 File Data underlying the revised Fig 4A blots. (JPG)

S2 File Quantitative data underlying the revised Fig 4A. (XLS)

S3 File Data underlying the Fig 2D blots. (JPG)

S4 File Quantitative data underlying Fig 2D. (XLS)
