# Supplementary figures and images for: Correction: Overexpression of Nrdp1 in the Heart Exacerbates Doxorubicin-Induced Cardiac Dysfunction in Mice
Source: PLoS One. 2022 Apr 19;17(4):e0267515. doi: 10.1371/journal.pone.0267515 (PMC9017932; doi:10.1371/journal.pone.0267515)

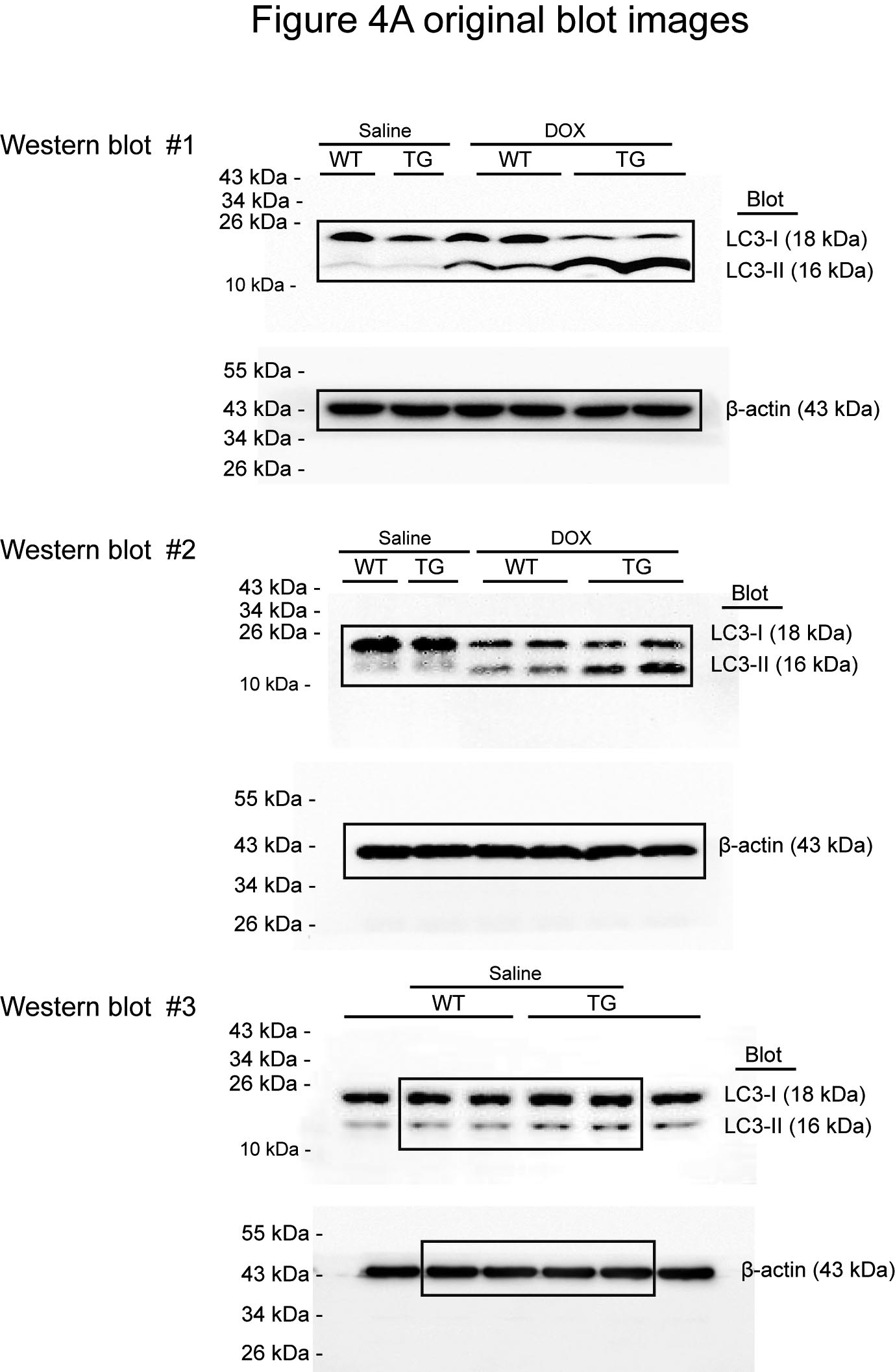

Supplement: S1 File — (JPG) [file pone.0267515.s001.jpg]

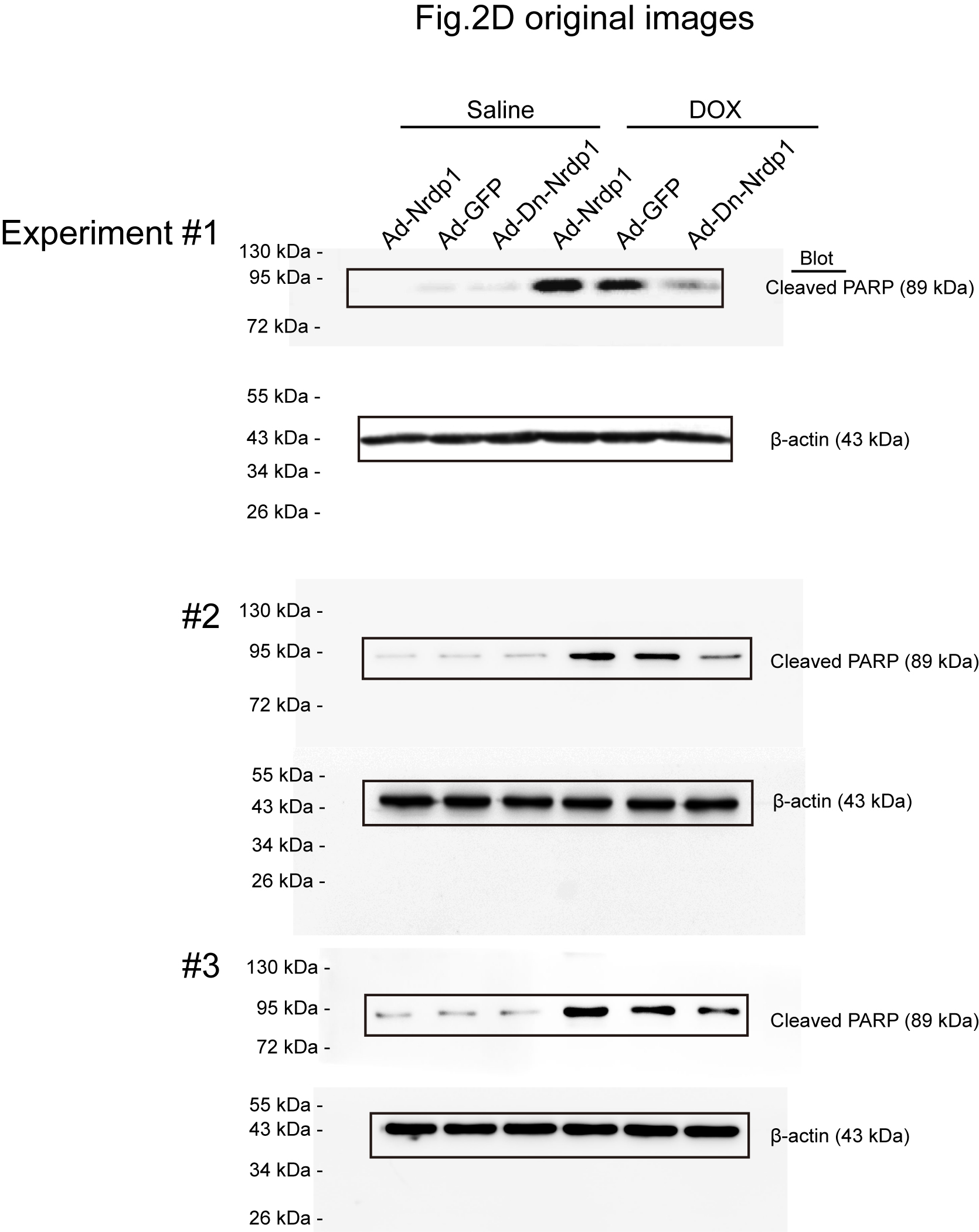

Supplement: S3 File — (JPG) [file pone.0267515.s003.jpg]
